# MLR and ANN Approaches for Prediction of Synthetic/Natural Nanofibers Diameter in the Environmental and Medical Applications

**DOI:** 10.1038/s41598-020-65121-x

**Published:** 2020-05-15

**Authors:** Saba Kalantary, Ali Jahani, Reza Jahani

**Affiliations:** 1 Department of Occupational Health Engineering, School of Public Health, Tehran University of Medical Sciences, Tehran, 1416753955 Iran; 2 Department of Natural Environment and Biodiversity, Faculty of Environment, College of Environment, Karaj, 31746118 Iran; 3 Department of Toxicology and Pharmacology, School of Pharmacy, Shahid Beheshti University of Medical Sciences, Tehran, 1416753955 Iran

**Keywords:** Mathematics and computing, Nanoscience and technology

## Abstract

Fiber diameter plays an important role in the properties of electrospinning of nanofibers. However, one major problem is the lack of a comprehensive method that can link processing parameters to nanofibers’ diameter. The objective of this study is to develope an artificial neural network (ANN) modeling and multiple regression (MLR) analysis approaches to predict the diameter of nanofibers. Processing parameters, including weight ratio, voltage, injection rate, and distance, were considered as independent variables and the nanofiber diameter as the dependent variable of the ANN model. The results of ANN modeling, especially its high accuracy (*R*^2^ = 0.959) in comparison with MLR results (*R*^2^ = 0.564), introduced the prediction the diameter of nanofibers model (PDNFM) as a comparative model for predicting the diameter of poly (3-caprolactone) (PCL)/gelatin (Gt) nanofibers. According to the result of sensitivity analysis of the model, the values of weight ratio, distance, injection rate, and voltage, respectively, were identified as the most significant parameters which influence PDNFM.

## Introduction

Gelatin (Gt), a natural biopolymer, which is derived from collagens, has been widely used in environmental and biomedical applications due to its biocompatible, biodegradability, and low cost^1,2^. Among the synthetic polymers, poly (3-caprolactone) (PCL) is a semicrystalline linear hydrophobic, biocompatible, and low-cost polymer, which has been widely used^3^. Different studies have shown that the polymer blend provides a compromised polymer solution for overcoming the shortcomings of synthetic and natural polymers, causing a new biomaterial with improving physical, mechanical and chemical properties and excellent biocompatibility^4^.

In recent years, electrospinning is recognized as a simple, cost-effective, unique, and efficient technique to fabricate continuous polymeric, ceramic, and hybrid nanofibers with narrow diameter distribution. Electrospinning has shown great potential due to its various applications in filter media, solar cell, drug delivery, tissue engineering, purification members, sound absorption, biosensor, wound dressing, and protective materials and can be applied in large scale production^5–7^. For improving the efficacy of electrospinning nanofibers, their morphology and properties such as mechanical, electrical, optical, and biomedical should be adjusted in the ranges. Among these, control of the size of electrospinning nanofibers is an inevitable approach in nanofibers application^8,9^. The diameter of nanofibers depends on electrospinning parameters including processing conditions (injection rate, applied voltage, tip to collector distance, etc.), polymer solution properties (viscosity, conductivity, polymer concentration, surface tension, etc.), and ambient conditions (relative humidity, temperature, and atmosphere pressure)^10,11^. Detecting the relation between the electrospinning parameters and the morphology and diameter of the obtained nanofibers is very difficult and time-consuming. It is better to apply a modeling approach to estimate and optimize the size of nanofibers before electrospinning^6^. Regression analysis, as one of the traditional tools, has been used in the model generation. Multiple regression (MLR) analysis is a statistical method, which explores the relation between independent and dependent variables^12–15^. The accuracy of the regression models increases by using MLR while it declines when independent variables increase. Nonlinear and dynamic modeling techniques like artificial neural network (ANN) are modeling tools to solve complex cases, quality control, data mining, and linear and nonlinear multivariate regression problems^16–19^. In recent years ANN approach as one of the most popular artificial intelligence approaches has been used to model the electrospinning technique, mostly aimed at predicting the diameter of nanofibers electrospinning^16,20^. The accuracy of multilayer perceptron artificial neural network (MLP) in comparison with other ANN techniques such as Radial Basis Function (RBF) and Support Vector Machine (SVM) in nanofibers diameter prediction has been proved in recent researches. Researchers declared that the reliable results of the ANN in nanofiber studies are in the complex interactions between the variables which are influencing nanofiber formation^21^. However, the capability of ANN techniques, in nanofibers diameter prediction, has not been compared with classic regression methods such as MLR. The objective of this research is to compare the classical regression method with a multilayer perceptron artificial neural network (MLP) for predicting the diameter of PCL/Gt nanofibers electrospinning and developing a probabilistic model to predict the diameter of PCL/Gt nanofibers (PDNF) using objective criteria.

## Material and Methods

### Materials

Gelatin, from porcine skin type A (Gel Strength _300 g Bloom), PCL (Mw = 80000 g/mol), glacial acetic acid, and formic acid were all purchased from Sigma-Aldrich.

### Preparation of polymer solution and electrospinning

A separate solution was prepared from PCL and gelatin by dissolving 15% w/w of the sample in glacial acetic acid/formic acid in a 9:1 ratio (AA/FA) via magnetic stirrer for 4 h. Following this, PCL and gelatin (PCL/Gt) were mixed at different weight ratios (80:20, 70:30, 60:40, 50:50, 40:60, 30:70, and 20:80) for 20 h prior to electrospinning. For electrospinning, each PCL/Gt solution was added in a 5 ml syringe with a needle tip (23 G). Electrospinning was carried out with an injection rate of 0.6–2 ml/h. The distance between the needle tip and the collector was 5–20 cm. The applied voltage was in the range of 6–22 kV. All experiments were conducted at room temperature^22,23^.

### Characterization

Morphology of nanofibres was observed under a scanning electron microscope (SEM, DSM-960A Model, ZEISS, Germany) at an accelerating voltage of 20 kV. Before SEM, samples were coated with gold. For each sample, the average fiber diameter determined from about 70 random measurements using Image J software.

### Artificial intelligence modeling

The ANN is known as one of the main tools in the modeling and control of electrospinning processes in recent years^24,25^. The ANN, as a computing tool, represents a network with several numbers of layers, including many interconnected processing elements (PEs), which are only aware of signals^26^. Indeed, ANNs are capable of learning from real samples of a problem, using transfer functions between neurons and specific learning algorithms in the structure of computer software^27–29^.

Four parameters, namely the applied voltage (X_1_,.kV), the injection rate of solution (X_2_, ml/h), the weight ratio of polymers (X_3_, wt%), and the needle-to-collector distance (X_4_, cm) were considered as input variables of the ANN and the average PCL/Gt nanofibers diameter (Y, nm) was chosen as the output. In this study, hyperbolic tangent, sigmoid tangent, and linear transfer functions were examined to optimize the performance of the neural network^30^. The backpropagation (BP) was applied as a learning algorithm for calculating derivatives of performance concerning the weight and bias variables X. To do an evaluation, all samples (761 samples) were randomly divided into three subsets. The training data set contained 60% of all samples (457 samples), the validation data set included 20% of all samples (152 samples), and test data set included 20% of all samples (152 samples). The validation data set is applied to decrease the possibility of over-fitting or memorizing. It means that when the error of the training data set decreases while the error of the validation data set increases, the network training process will be stopped and over-fitting will be controlled^28,31^. ANN may be trapped in a local minimum of errors and the Momentum coefficient helps to avoid local minimum error traps. Therefore the possibility of under-fitting will be reduced by using the Momentum coefficient. In this research, the Momentum coefficient, initial momentum, and learning rate are 0.9, 0.001, and 0.01 respectively. Levenberg- Marquardt (LM) learning algorithm was used to train the network. This algorithm solves generic curve-fitting problems, but the LM maybe is trapped in a local minimum. Therefore, the momentum coefficient was assigned to avoid the local minimum trap. The Levenberg- Marquardt is more robust than other algorithm and in many cases it results in the best performance of the network^32,33^.

To design the structure of feed-forward and back-forward networks, a program was provided in MATLAB software (Version R2016b). There is not any predefined rule to determine the number of neurons and layers in the structure of ANN, therefore the number of neurons and layers are defined based on trial and error^28,34^. In this study in order to reduce output error, the number of neurons and layers increased and after that, any increase in the number of neurons and layers does not increase the accuracy of the model

### Model selection

The performance of the designed ANN was evaluated by different statistical indicators: mean squared error – MSE (Eq. 1), root mean squared error – RMSE (Eq. 2), mean absolute error – MAE (Eq. 3), coefficient of determination – R^2^ (Eq. 4) and Nash–Sutcliffe model efficiency coefficient (NSE) (Eq. 5)^35^.

1 $$MSE=\frac{{\sum }_{i=1}^{n}{({y}_{i}-{\hat{y}}_{i})}^{2}}{n}$$

2 $$RMSE=\sqrt{\frac{{\sum }_{i=1}^{n}{({y}_{i}-{\hat{y}}_{i})}^{2}}{n}}$$

3 $$MAE=\frac{{\sum }_{i=1}^{n}|{y}_{i}-{\hat{y}}_{i}|}{n}$$

4 $${R}^{2}={\left(\frac{{\sum }_{i=1}^{n}({y}_{i}-{\bar{y}}_{i})({\hat{y}}_{i}-{\overline{y^{\hat{}}}}_{i})}{\sqrt{{\sum }_{i=1}^{n}{({y}_{i}-{\bar{y}}_{i})}^{2}}\sqrt{{\sum }_{i=1}^{n}{({\hat{y}}_{i}-{\overline{y^{\hat{}}}}_{i})}^{2}}}\right)}^{2}$$

5 $$NSE=1-\frac{{\sum }_{i=1}^{n}{({\hat{y}}_{i}-{y}_{i})}^{2}}{{\sum }_{i=1}^{n}{({y}_{i}-{\bar{y}}_{i})}^{2}}$$

where ${y}_{i}$ and ${\hat{y}}_{i}$ are the targets and network outputs, ${\bar{y}}_{i}$ is the mean of target values, ${\overline{y^{\hat{}}}}_{i}$ is the mean of output values, and n is the number of samples, respectively.

Sensitivity analysis was conducted to rank prediction the diameter of PCL/Gt nanofibers model (PDNFM) parameters considering the significance of each parameter in the model output.

### Sensitivity analysis of the model

For the analysis of the importance of each electrospinning parameters, each input parameter was withdrawn while not manipulating any of the other parameters. Then the model was trained for every pattern. It means that the standard deviation was calculated for each input variable and the changes of each input around the mean value (in the limits of standard deviation) used to determine the changes of output. Indeed, the standard deviation of output values for each input variable changes assigned as the sensitivity of the model. The changes in model output values with changes in input variables (in the limits of standard deviation) illustrate the trend of the model^21^.

## Results

In this research, two predictive models, i.e., MLR analysis and ANN model, were investigated to compare findings in PDNF model prediction.

### MLR model

Four independent variables (needle-to-collector distance, the injection rate of the polymer solution, weight ratio, and applied voltage) were used to predict the diameter of nanofibers. To avoid any possible bias in the selection of test set individuals, the total samples (761 samples) were randomly divided into two subsets. Training data subset including 80% of total samples (609 samples), and test data subset, including 20% of total samples (152 samples). Using the training data subset, constant coefficients of the regression equation were calculated, while the summation of square errors was minimized. Then the prediction operation was carried out on test data – 20% of samples (152 samples). Equation 6 was used to predict the PDNFM:

6 $$\begin{array}{cc}PDNFM= & 800.514-(6.372\times PCL\,weigh\,ratio)-(8.212\times D)-(3.426\times V)\\  & +\,(100.224\times injection\,rate)\end{array}$$

where D and V are the distance between needle to collector and voltage, respectively.

Statistical indices were calculated to estimate the MLR model’s accuracy in the prediction of PDNFM, and the findings are illustrated in Table 1.

**Table 1:** Statistical indices of multiple regression model in training and test sets.

Performance measures	set			All data
	Training	test		
R^2^	0.5667		0.5551	0.564
MSE (nm)	15634		18049	16116
RMSE (nm)	125		134	126
MAE (nm)	90.9653		95.7051	91.9120
NSE	0.567		0.554	0.564

**Figure 1:**
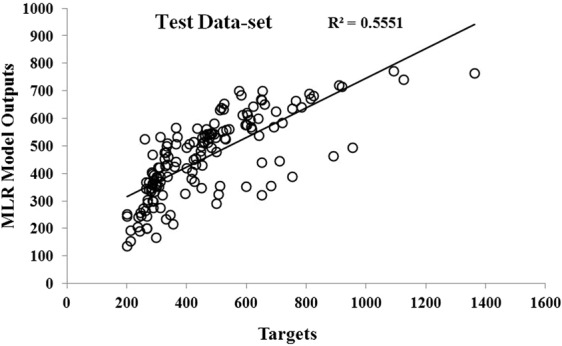
The scatter plot of target versus predicted the diameter of nanofibers model (PDNFM) by multiple regression (MLR) in test samples.

The relation between target and predicted PDNFM by MLR model had been plotted using a linear regression model (Fig. 1).

### Artificial intelligence modeling

Affecting parameters on PDNFM as inputs variables, and PDNFM as output were summarized in the MATLAB software for the design of the most accurate structure of ANN. The data provided from affecting parameters were applied to train the feedforward neural networks. The maximum value of R^2^ in all data considered (Table 2). The best ANN structure is (4-28-28-1), which means 4 parameters as inputs, 28 neurons in the first hidden layer, 28 neurons in the second hidden layer and one neuron in the output layer, respectively. The Sigmoid tangent transfer function was applied by the hidden and output layers.

**Table 2:** The performance measures of the best artificial neural networks.

Performance measures	set				All data
	training	validation		test	
R^2^	0.9679	0.9481	0.9452		0.9594
MSE (nm)	1188	1723	2221		1501
RMSE (nm)	34.47	41.51	47.13		38.74
MAE (nm)	26.38	31.71	33.58		28.88
NSE	0.9679	0.948	0.9451		0.9594

Equation 7 represents the structure of calculations in optimized MLP. However, the biases and the weights for neurons and layers are summarized in huge matrixes that are saved in the structure of the network in MATLAB software.

7 $${\rm{P}}{\rm{D}}{\rm{N}}{\rm{F}}{\rm{M}}={\rm{t}}{\rm{a}}{\rm{n}}{\rm{s}}{\rm{i}}{\rm{g}}({\rm{t}}{\rm{a}}{\rm{n}}{\rm{s}}{\rm{i}}{\rm{g}}(\sum {\rm{L}}{{\rm{W}}}_{2,1}{\rm{t}}{\rm{a}}{\rm{n}}{\rm{s}}{\rm{i}}{\rm{g}}(\sum {\rm{I}}{{\rm{W}}}_{1,1{\rm{p}}{\rm{i}}}+{\rm{b}}1)+{\rm{b}}2))$$

In which, p_i_ is input layer values, IW_ji_ is the weight of neurons, LW_ji_ is the weight of layers, b_i_ is bias, and tansig is the sigmoid tangent function (tansig(X)= 2/(1+exp(−2*x))−1). As we know, IW_ji_ and LW_ji_ are structured in a huge matrix of weights, which is applicable in MATLAB software. Therefore, this model is calculable in MATLAB software by running the designed network^32,36,37^.

**Figure 2:**
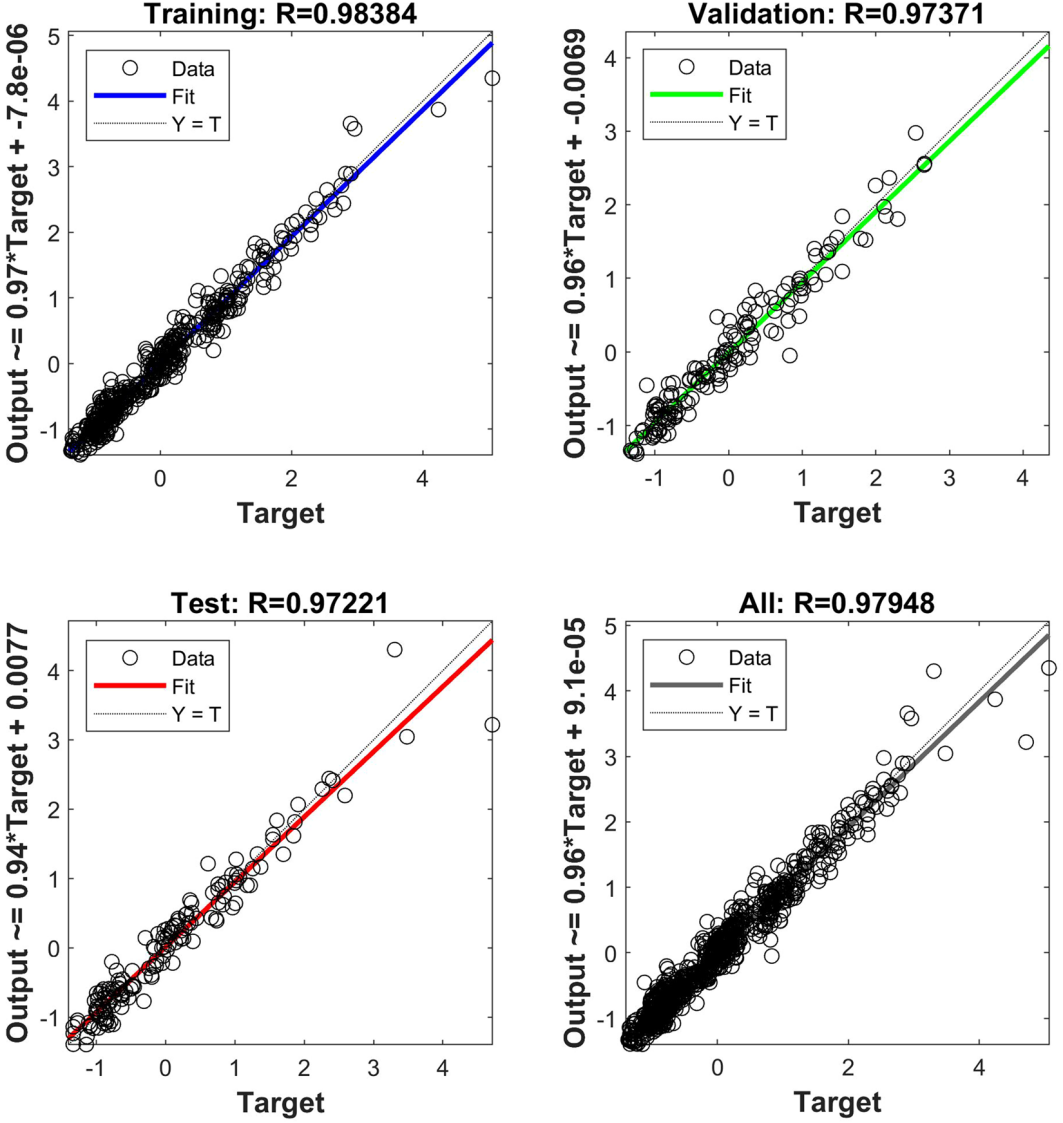
Scatter plots of output versus target predict the diameter of PCL/Gt nanofibers values by artificial neural network.

The scatter plot will be applicable to demonstrate the correlation between variables^38^. Figure 2 provides the scatter plot of ANN output versus target (observed) values of the PDNFM for training, validation, test, and all data. Considering *R*^2^, the correlation coefficient between the ANN output and target values of PDNFM is relatively high.

**Figure 3:**
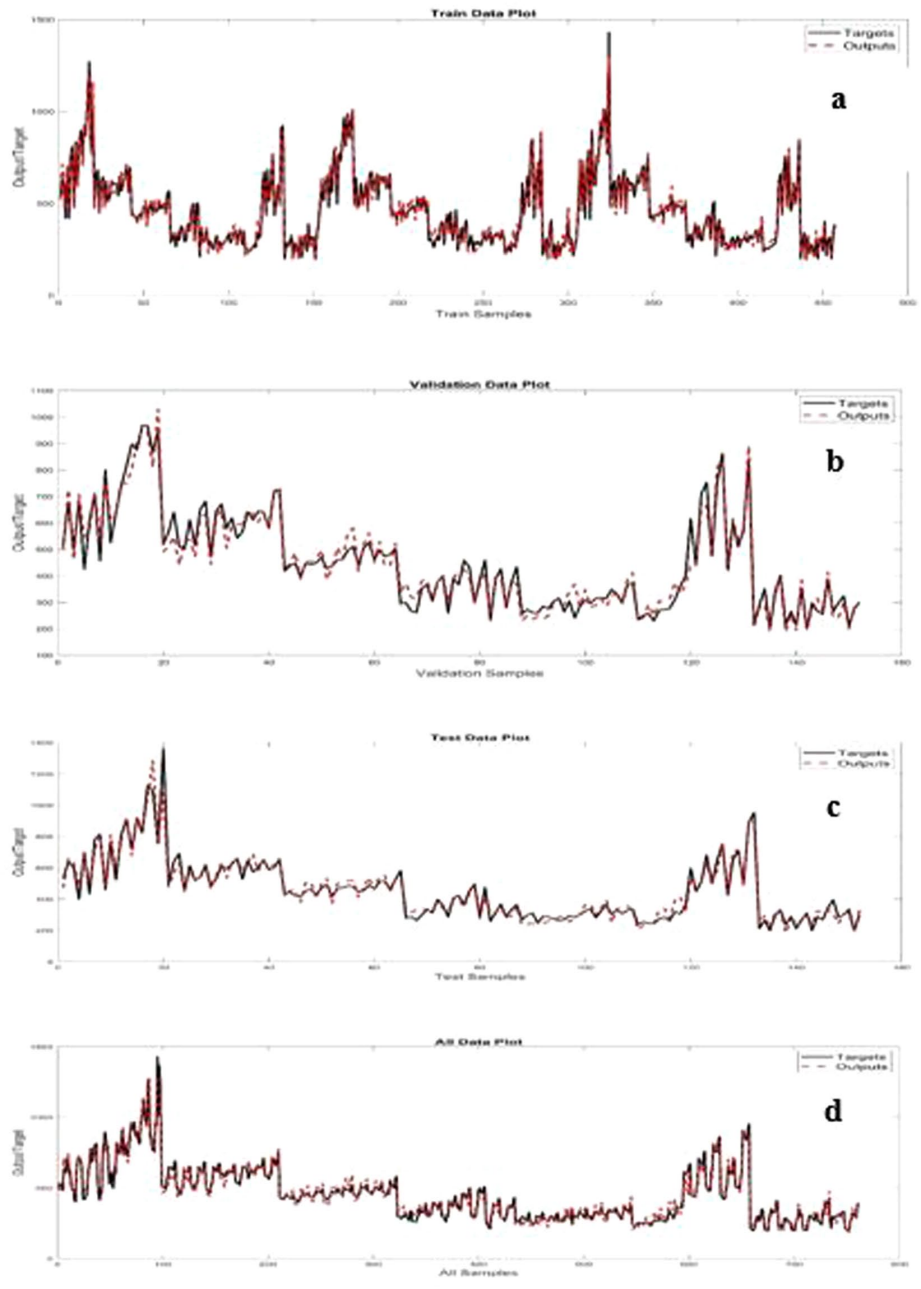
Target and simulated diameter of nanofibers values by artificial neural network: training data set (**a**), validation data set (**b**), test data set (**c**), all data (**d**).

Figure 3 compares the target and simulated values of PDNFM in the training, validation, test data set, and all data. A meaningful and distinctive agreement between target and simulated values is shown in Fig. 3.

**Figure 4:**
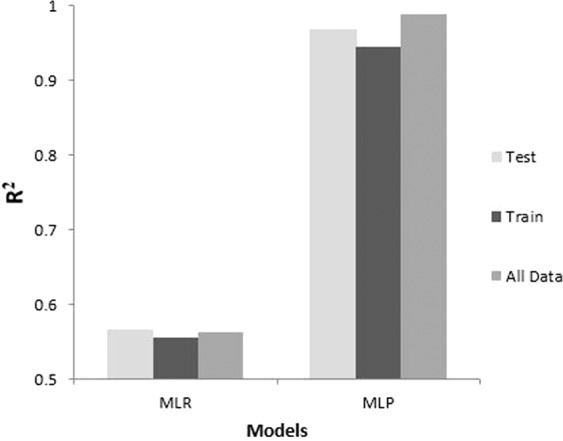
The performance measures of the designed PDNFM.

The main application of PDNFM in which it’s used to predict the nanofibers size based on electrospinning processing parameters. This model could be applied as a decision support system tool in predicting the diameter of electrospinning nanofibers to reduce the time and costs. The compare findings of PDNFM_MLR_ and PDNFM_MLP_ show that the PDNFM_MLP_ is the most accurate model in the prediction of the diameter of PCL/Gt electrospinning nanofibers (Fig. 4).

### Sensitivity analysis of PDNFM

**Figure 5:**
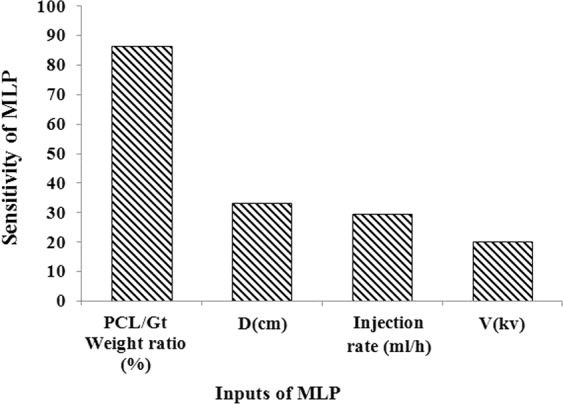
Results of the sensitivity analysis of PDNFM.

For the analysis of the importance of each electrospinning parameters, each input parameter was withdrawn while not manipulating any of the other parameters, and then the PDNFM was trained for every pattern. As can be seen from Fig. 5, the share of each input parameter of the developed PDNFM in favorable output can be understood clearly. From the data obtained from the sensitivity analysis model, it is apparent that values of the PCL/Gt weight ratio, the needle-to-collector distance, the injection rate polymer solution, and applied voltage, respectively, have been recognized as the critical factors for PDNFM (Fig. 5).

**Figure 6:**
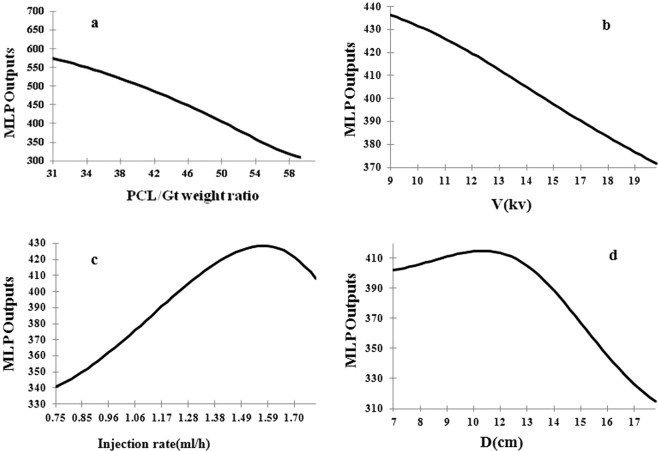
Prediction the diameter of nanofibers model (PDNFM) output for inputs parameters: (**a**) PCL/Gt weight ratio (**b**) applied voltage (**c**) injection rate (**d**) distance.

The effect of electrospinning processing parameters on MLP outputs (the diameter of PCL/Gt nanofibers) is shown in Fig. 6.

## Discussion

In this investigation, the main goal was to assess which modeling technique has better accuracy. Therefore, the experimental and predicted data obtained by MLR analysis and MLP model were evaluated to obtain an accurate fit. Consequently, error analysis was used, and R^2^, MSE, RMSE, and MAE were calculated. The resulting MLP model with R^2^ = 0.959 is in perfect agreement with experimental results than the R^2^ value found in the MLR analysis.

The MLR analysis has a low R^2^ value, meaning that it has a lower accuracy than the MLP model. If the correlation coefficient threshold is calculated one, it represents a perfect correlation between targets and output values of the training/testing data^39–41^. The results obtained of the ANN modeling, especially its high accuracy (*R*^2^ = 0.959) in comparison with MLR results (*R*^2^ = 0.564), introduced PDNFM_mlp_ as a comparative model for predict the diameter of PCL/Gt nanofibers.

This fact indicates that the MLP model can be more predictive accuracy. However, these values were satisfactory because the electrospinning process and the diameter size of electrospinning nanofibers have high degrees of complexity^9^. The evidence showed in previous studies suggests similar findings^42–44^. Nurwaha and Wang (2013) compared the neuro-fuzzy inference systems (ANFIS) and support vector machines (SVMs), an MLR for evaluation of electrospinning nanofibers diameter. Taken together, the evidence from this research presents that the performance of the SVM model was better than ANFIS and MLR techniques. Accordingly, the values of RMSE and MAE for the SVM are 8.21 and 6.56, for the ANFIS are 9.98 and 8.89, and for MLR are 19.73 and 15.78, respectively^43^. To determine the effects of the content of poly(butylene adipate) and teriflunomide on an initial burst effect and a dissolution behavior, Siafaka *et al*. (2016) has raised compared ANN and MLR models. The ANN model was more accurate and it had better correlation efficacy compare to MLR analysis. The R^2^ value for the MLR and ANN model is 0.85 and 0.945, respectively^42^. Vle *et al*. (2015) measured the physical properties of nylon-6 fibers and compared them with measured values based on MLR and ANN models. Considering all relevant data, it seems that the ANN model can be applied efficiently in predicting the physical properties of fibers. The ANN model showed well correlation and provided stable responses comparison to MLR. Overall, these results indicated that the ANN model would very useful for predicting combined interaction between independent variables^44^. The ANN techniques provide the advantage of modeling a nonlinear and complicated problem without the need to find suitable functional forms for the problem, and their neural network learning ability also equips them with high efficiency in nonlinear system modeling^43,45^. Together, these studies indicate that ANNs techniques carried out well and illustrated stable responses in predicting combined interactions between independent parameters^42–44^.

The present study explores the effect of injection rate polymer solution, applied voltage, PCL/Gt weight ratio, and tip to collector distance on the average nanofiber diameter. The finding of sensitivity analysis found that there are close relationship processing parameters and MLP output. From sensitivity analysis results (see Fig. 5), PCL/Gt weight ratio parameter has a highly effect on the average nanofiber diameter. It is observed that the fiber diameter was decreased, by increasing the content of PCL in the AA/FA solution and applied voltage (see Fig. 6 (a) and (b)), as a result, there is a reverse correlation between the applied voltage and weight ratio polymer and fiber diameter. Increasing PCL content will result in lower polymer solution emulsion (Fig. 6(a)). The evidence presented in other studies suggests that the average diameter decreases with PCL content for acetic acid or acetic acid/formic acid mixture system as a solvent. This emulsion structure is related to absence, or very limited miscibility, PCL and Gt, and the interaction of weak PCL and Gt with AA and FA. This means that at higher PCL content, the emulsion structure is weakened^22^. These results are in agreement with Denis *et al*.^’^s finding^22^. Furthermore, the viscosity of polymer solution decrease with an increase in the PCL content of the polymer solution blend. Accordingly, thinner fiber formed due to that the jet could be stretched by electrostatic forces easily^46,47^. Considering trends in Fig. 6(b), applied voltage in electrospinning PCL/Gt is negatively correlated with the average diameter. At high applied voltage, the electrical field strength is high, resulting in more stretching jet during the jet path, and hence, it is expected that the nanofibers diameter decrease^46^. In general, decreasing fiber size is due to the fact that the surface of charge on the jet at higher voltage or field increased. This observation is similar to the previously published reports^21,48,49^. Figure 6(c) provides the effect of the injection rate on the diameter of nanofibers. As one can see, the diameter of nanofibers increases as well as decreases with the increase of the injection rate. The previous investigations suggest similar results^9,50^. With the increasing injection rate, it is expected that the nanofiber’s diameter increases. Accordingly, an increase in the diameter of nanofibers was obtained with an increase in the injection rate of polymer solution due to the increases the amount of polymer solution on the tip of the needle^9,46,49,50^. However, when the injection rate exceeds a certain limit increase, the diameter of the nanofiber continuously decreased. Increasing, the injection rate will result in the higher electrical field, increase in the volumetric charge density on the droplet jet, and greater tensile force which this phenomenon creates stretching during jet path and hence the diameter of nanofibers will decrease^5,48^. One interesting findings demonstrated here indicate that distance has a double effect on the nanofibers size (Fig. 6(d)). The increase in the distance between needle and collector was accompanied by an increase in the size of nanofibers, but electrospinning distance more than a certain value exhibited a decrease in the diameter of nanofibers. This behavior is explained by the decrease in solvent evaporation time, before nanofibers deposited on collector versus, the diameter fiber reduce with increasing the distance owing to that solvent evaporation time increased and jet stretched before deposited on the collector^51^. A review of other studies reported that an increase in the distance between needle and collector causes a decrease in the nanofibers size. This is probably owing to breaking the formed jet into two or more jets, leading to finer nanofibers^9^. With regard to the recent progress in electrospinning, the findings suggest that modeling methods such as ANN techniques can be important implications for controlling and prediction the diameter of electrospinning nanofibers, which is a critical factor in determining the properties of nanofibers.

## Conclusions

In this research, the application of the multiple regression analysis and MLP model was studied to predict the electrospinning PCL/Gt nanofiber diameter. The finding of this study suggests that an ANN technique can be used quite effectively for prediction the diameter of nanofibers. The main application of PDNFM_MLP_ is to predict the diameter of nanofibers based on electrospinning processing parameters. As a decision support system tool, PDNFM could assist researchers, engineers, and expert’s lab in fabricating electrospinning nanofibers with defined fiber diameter. It can be worthwhile in the aspect of economic, time, and scientific aims. However, it is interesting to note that the effects of electrospinning parameters are highly depended on the type of polymer used. Also, it is suggested that future research, which takes more parameters into account, will need to be undertaken with higher accuracy over a more extensive application range.

## Data Availability

This article has no additional data.

## References

[1] Maleknia L, Majdi ZR (2014). Electrospinning of gelatin nanofiber for biomedical application. Orient j. chem..

[2] Powell HM, Boyce ST (2008). Fiber density of electrospun gelatin scaffolds regulates morphogenesis of dermal–epidermal skin substitutes. Journal of Biomedical Materials Research Part A: An Official Journal of The Society for Biomaterials, The Japanese Society for Biomaterials, and The Australian Society for Biomaterials and the Korean Society for Biomaterials..

[3] Binulal NS (2014). PCL–gelatin composite nanofibers electrospun using diluted acetic acid–ethyl acetate solvent system for stem cell-based bone tissue engineering. *J*. Biomater Sci Polym Ed..

[4] Chong EJ (2007). Evaluation of electrospun PCL/gelatin nanofibrous scaffold for wound healing and layered dermal reconstitution. Acta Biomater..

[5] Paskiabi FA (2015). Optimizing parameters on alignment of PCL/PGA nanofibrous scaffold: An artificial neural networks approach. *Int*. J. Biol. Macromol..

[6] Karimi MA (2015). Using an artificial neural network for the evaluation of the parameters controlling PVA/chitosan electrospun nanofibers diameter. e-Polymers..

[7] Beigzadeh Z, Golbabaei F, Khadem M, Shahtaheri SJ (2019). Fabrication and Optimization of Molecularly Imprinted Nanofibers in Assessment of Occupational Exposure to 5-fluorouracil. J Mazandaran Univ Med Sci..

[8] Esnaashari SS, Naghibzadeh M, Adabi M, Faridi Majidi R (2017). Evaluation of the Effective Electrospinning Parameters Controlling Kefiran Nanofibers Diameter Using Modelling Artificial Neural Networks. Nanomed Res J..

[9] Faridi‐Majidi R, Ziyadi H, Naderi N, Amani A (2012). Use of artificial neural networks to determine parameters controlling the nanofibers diameter in electrospinning of nylon‐6, 6. J. Appl. Polym. Sci..

[10] Nasouri K, Bahrambeygi H, Rabbi A, Shoushtari AM, Kaflou A (2012). Modeling and optimization of electrospun PAN nanofiber diameter using response surface methodology and artificial neural networks. J. Appl. Polym. Sci..

[11] Nasouri K (2018). Novel estimation of morphological behavior of electrospun nanofibers with artificial intelligence system (AIS). Polym Test..

[12] Jahani A (2019). Forest landscape aesthetic quality model (FLAQM): A comparative study on landscape modelling using regression analysis and artificial neural networks. J. For. Sci..

[13] Jahani A (2017). Aesthetic quality evaluation modeling of forest landscape using artificial neural network. Wood & Forest Science and Technology.

[14] Akbarifard S, Radmanesh F (2018). Predicting sea wave height using Symbiotic Organisms Search (SOS) algorithm. Ocean Eng..

[15] Alefi M, Sadeghi Yarandi M, Karimi A (2020). Modeling of Occupational Risk Factors in the Development of Musculoskeletal Disorders in Nurses. Archives of Occupational Health..

[16] Vatankhah E (2014). Artificial neural network for modeling the elastic modulus of electrospun polycaprolactone/gelatin scaffolds. Acta Biomater.

[17] Jahani A, Mohammadi FA (2017). Aesthetic quality modeling of landscape in urban green space using artificial neural network. Journal of Natural Environment.

[18] Qaderi K, Akbarifard S, Madadi MR, Bakhtiari B (2017). Optimal operation of multi-reservoirs by water cycle algorithm. P I Civil Eng-Wat M Journal.

[19] Yang, X., Gandomi, A. H., Talatahari, S. & Alavi, A. H. Metaheuristics in Water, *Geotechnical and Transport Engineering*, 231–270 (Elsevier, 2013).

[20] Khanlou HM, Ang BC, Barzani MM (2016). Prediction, modeling and characterization of surface texturing by sulfuric etchant on non-toxic titanium bio-material using artificial neural networks and fuzzy logic systems. Sci Eng Compos Mater..

[21] Kalantary S, Jahani A, Pourbabaki R, Beigzadeh Z (2019). Application of ANN modeling techniques in the prediction of the diameter of PCL/gelatin nanofibers in environmental and medical studies. RSC Adv..

[22] Denis P, Dulnik J, Sajkiewicz P (2015). Electrospinning and structure of bicomponent polycaprolactone/gelatin nanofibers obtained using alternative solvent system. INT J PolymMater Po Journal.

[23] Dulnik J, Denis P, Sajkiewicz P, Kołbuk D, Choińska E (2016). Biodegradation of bicomponent PCL/gelatin and PCL/collagen nanofibers electrospun from alternative solvent system. Polym Degrad Stabil..

[24] Aghajani H (2014). Investigation of affective habitat factors affecting on abundance of wood macrofungi and sensitivity analysis using the artificial neural network (case study: Kheyrud forest, Noshahr). Iranian Journal of Forest and Poplar Research..

[25] Akbarifard S, Bakhtiari B (2018). Optimal allocation of water resources using Water Cycle Algorithm (WCA)(Case study: Gorganrood basin). Water Eng..

[26] Jahani A (2017). Sycamore failure hazard risk modeling in urban green space. Journal of Spatial Analysis Environmental Hazarts..

[27] Jahani A (2019). Sycamore failure hazard classification model (SFHCM): an environmental decision support system (EDSS) in urban green spaces. Int J Sci Environ Technol.

[28] Jahani A, Feghhi J, Makhdoum MF, Omid M (2016). Optimized forest degradation model (OFDM): an environmental decision support system for environmental impact assessment using an artificial neural network. J Environ Plann Man.

[29] Das SK (2013). 10 Artificial Neural Networks in Geotechnical Engineering: Modeling and Application Issues. Metaheuristics in Water Geotech Transp Eng..

[30] Jahani A (2016). Modeling of forest canopy density confusion in environmental assessment using artificial neural network. Iranian Journal of Forest and Poplar Research..

[31] Hagan MT, Demuth HB, Jesus OD (2002). An Introduction to the Use of Neural Networks in Control Systems. Int J Robust Nonlin.

[32] Das SK, Basudhar PK (2008). Prediction of residual friction angle of clays using artificial neural network. Eng Geol..

[33] Das SK, Basudhar PK (2006). Undrained lateral load capacity of piles in clay using artificial neural network. Comput Geotech..

[34] Arsene CTC, Gabrys B, Al-Dabass D (2012). Decision Support System for Water Distribution Systems Based on Neural Networks and Graphs Theory for Leakage Detection. Expert Syst Appl.

[35] Samiei S, Pourbabaki R (2019). Risk Factors of Low Back Pain Using Adaptive Neuro-Fuzzy. Archives of Occupational Health.

[36] Chebrolu A, Sasmal SK, Behera RN, Das SK (2020). Prediction of Factor of Safety For Slope Stability Using Advanced Artificial Intelligence Techniques. Advanced Engineering Optimization Through Intelligent Techniques.

[37] Das SK, Basudhar PK (2007). Prediction of hydraulic conductivity of clay liners using artificial neural network. Lowl. Technol. Int..

[38] Flott LW (2012). Using the scatter diagram tool to compare data, show relationship. Metal Finishing.

[39] Khanlou HM (2014). Prediction and optimization of electrospinning parameters for polymethyl methacrylate nanofiber fabrication using response surface methodology and artificial neural networks. Neural Comput Appl..

[40] Samadian H (2016). Effective parameters on conductivity of mineralized carbon nanofibers: an investigation using artificial neural networks. RSC Advances.

[41] Sadollah A, Ghadimi A, Metselaar IH, Bahreininejad A (2013). Prediction and optimization of stability parameters for titanium dioxide nanofluid using response surface methodology and artificial neural networks. Sci Eng Compos Mater.

[42] Siafaka PI, Barmbalexis P, Bikiaris DN (2016). Novel electrospun nanofibrous matrices prepared from poly (lactic acid)/poly (butylene adipate) blends for controlled release formulations of an anti-rheumatoid agent. Eur. J. Pharm. Sci..

[43] Nurwaha D, Wang X (2013). The Use of Adaptive Neuro-Fuzzy Inference Systems and Support Vector Machines Techniques for Evaluation of Electrospun Nanofiber Diameter. J Comput Theor Nanos..

[44] Vle NFL, Najlona EVI, Umetne ZUM (2015). Predicting the Physical Properties of Drawn Nyylon-6 Fibers Using an Artificial- Neural- Network Model. Materiali in tehnologije.

[45] Maleki, M. Artificial neural network prosperities in textile applications. In Artificial Neural Networks-Industrial and Control Engineering Applications. IntechOpen. 2011.

[46] Nurwaha D, Han W, Wang X (2013). Effects of processing parameters on electrospun fiber morphology. J. Tex. I..

[47] Baghersad S, Bahrami SH, Mohammadi MR, Mojtahedi MRM, Milan PB (2018). Development of biodegradable electrospun gelatin/aloe-vera/poly (ε-caprolactone) hybrid nanofibrous scaffold for application as skin substitutes. Mater. Sci. Eng. C..

[48] Theron S, Zussman E, Yarin A (2004). Experimental investigation of the governing parameters in the electrospinning of polymer solutions. Polymer..

[49] Thompson CJ, Chase GG, Yarin AL, Reneker DH (2007). Effects of parameters on nanofiber diameter determined from electrospinning model. Polymer.

[50] Shao Q, Rowe RC, York P (2006). Comparison of neurofuzzy logic and neural networks in modelling experimental data of an immediate release tablet formulation. Eur. J. Pharm. Sci..

[51] Wu, T. *et al*. Fabrication of shish-kebab-structured carbon nanotube/poly (ε-caprolactone) composite nanofibers for potential tissue engineering applications. *Rare Metals*,1–9, 10.1007/s12598-017-0965-y (2019).

